# Wearable Camera-Based Objective Screen Time and Its Combined Associations with Dietary and Physical Activity Factors in Relation to Childhood Obesity

**DOI:** 10.3390/nu17182990

**Published:** 2025-09-18

**Authors:** Yi Liu, Ke-Xin Wang, Yu-Xi Zhou, Shi-Yu Yan, Antje Hebestreit, Hai-Jun Wang

**Affiliations:** 1 Department of Maternal and Child Health, School of Public Health, Peking University, National Health Commission Key Laboratory of Reproductive Health, Beijing 100191, China; liuyi627@bjmu.edu.cn (Y.L.); yan_sy@bjmu.edu.cn (S.-Y.Y.); 2 Peking University Health Science Center-Weifang Joint Research Center for Maternal and Child Health, Beijing 100191, China; 3 Department of Computer Science, Tianjin University of Technology, Tianjin 300384, China; wangkexin314@stud.tjut.edu.cn (K.-X.W.); yuxi_mail@tsinghua.edu.cn (Y.-X.Z.); 4 DCST, BNRist, RIIT, Institute of Internet Industry, Tsinghua University, Beijing 100084, China; 5 Leibniz Institute for Prevention Research and Epidemiology-BIPS, 28359 Bremen, Germany; hebestr@leibniz-bips.de

**Keywords:** screen time, childhood obesity, wearable cameras, dietary behavior, physical activity

## Abstract

**Background and objectives**: The objective of this study was to test the effects of objectively measured screen time using wearable cameras, along with dietary intake and physical activity level (and their interaction), on obesity among Chinese school-aged children. **Methods**: This study was conducted among 52 fourth-grade children (age: 9.76 ± 0.44; 50% boy) in Beijing, including children with obesity and age- and sex-matched normal-weight controls. Screen time (min/day) was coded from wearable camera images collected over one week using image recognition, physical activity measured using accelerometers, and dietary intake via camera-assisted 3-day 24 h dietary recalls. Logistic regression and generalized additive models assessed associations with obesity indicators, including general (obesity; percentage of body fat, BF%) and central (waist circumference; weight-to-height ratio). The combined effects of screen time with dietary and physical activity factors were also analyzed. **Results**: Children with obesity had longer daily screen time (94.91 ± 35.44 vs. 83.15 ± 36.86 min). Longer screen time was associated with higher energy and carbohydrate intake, more average duration per meal, a higher proportion of meals with screen, increased sedentary time, and a lower proportion of time spent in moderate-to-vigorous physical activity (MVPA). After adjusting for dietary intake and demographic covariates, longer screen time (≥1.3 h/day) was linked to higher odds of obesity (OR = 4.25, 95% CI = 1.09, 16.53) and BF% (OR = 6.14, 95% CI = 1.29, 29.10). Less protein intake (OR = 9.57, 95% CI = 1.31, 70.14), more proportion of meals with screen (OR = 6.40, 95% CI: 1.22, 33.61), less proportion of meals with social interaction (OR = 5.90, 95% CI: 1.01, 34.59), and less MVPA (OR = 5.21, 95% CI = 1.11, 24.43) with more screen time increased obesity risk. **Conclusions**: Objectively measured screen time was positively associated with the risk of childhood obesity. Longer screen time combined with lower protein intake, a higher proportion of meals consumed while watching screens, less meals with social interaction, and lower MVPA may collectively increase obesity risk. These findings call for efforts to reduce screen exposure, promote MVPA, and increase dietary protein intake. Additionally, encouraging children to avoid screen use during meals and promoting parent or peer companionship during eating may help reduce the risk of childhood obesity.

## 1. Introduction

Unhealthy behaviors, such as increased screen time and prolonged sedentary behavior in children, are linked to a higher risk of obesity [1,2,3]. Screen time refers to the time spent using electronic devices such as televisions, computers, smartphones, and tablets [4]. Due to overall insufficient and inconsistent evidence, the World Health Organization (WHO) has not established specific daily screen time recommendations for children and adolescents aged 5 to 18 years [5]. However, the WHO has issued guidelines on overall sedentary behavior, which includes screen-related activities, recommending that it should be restricted to no more than 2 h per day. Among 9- to 10-year-olds in the United States and China, average daily screen time approached 4 h [6,7]. In the European IDEFICS/I.Family cohort, the average daily screen time for this age group was approximately 3.5 h [8]. Therefore, the screen time habits of school-aged children have become a growing public health concern. Both in China and internationally, excessive screen time among children of this age group requires urgent attention.

Screen time has been linked to unhealthy dietary intakes, which are in turn associated with an increased risk of obesity and metabolic syndrome in early life [8]. Television viewing time and overall screen time have been shown positively correlated with adverse dietary behaviors, including decreased consumption of fruits and vegetables, increased intake of unhealthy foods, and higher consumption of cariogenic items such as energy-dense snacks and sugar-sweetened beverages [9]. Furthermore, prolonged screen time was typically associated with extended sedentary behavior, which may reduce physical activity levels and overall energy expenditure, thereby contributing to a dual risk of increased energy intake and decreased energy expenditure. The formation of screen-related behaviors in children was influenced by the family context [10]. Excessive screen time has been associated with reduced parent–child interactions, as screen exposure in young children may hinder opportunities for verbal communication and meaningful engagement within the family context [11]. However, previous research has rarely examined the relationship between prolonged screen time and specific dietary habits, such as screen exposure during meals or eating in the presence of family or peers. The combined impact of these dietary behaviors and screen time on childhood obesity remains insufficiently explored.

At the same time, many studies have focused on the association between a single type of screen time and childhood obesity, which limited the ability to capture the overall impact of total screen time on obesity [12]. Additionally, prior studies have been constrained by methodological limitations, potentially leading to underreporting of actual screen time exposure among children. Most studies have relied on self- or parent-reported measures, which are inherently subjective and susceptible to recall bias [5,12], particularly among children and adolescents who may have limited cognitive development and are more prone to memory inaccuracies [13]. Self-reported questionnaires often fail to accurately capture the actual duration children spend on screens [14]. Moreover, such approaches rarely differentiate between device types (e.g., television, phone, computer, and tablet) or capture behaviors that occur simultaneously with screen use, such as eating or engaging in social interaction. Therefore, the use of objective measurement tools is necessary to obtain accurate assessments of children’s screen time exposure.

Emerging objective measurement tools, such as wearable cameras, have emerged as promising methods for capturing screen behaviors and quantifying screen time. Previous studies have utilized wearable cameras to analyze screen-related behaviors, revealing that children spend over a third of their time engaged in screen activities, with high screen use occurring predominantly during after-school and weekend periods [15,16]. These findings demonstrate the potential of wearable cameras to provide objective and accurate assessments of screen time. However, previous research relied on manual coding, which is labor-intensive and time-consuming, thereby limiting the scalability and applicability of this approach in large-scale population studies. By integrating image recognition techniques, such as deep learning models based on convolutional neural networks (CNNs) and residual networks (ResNets), more detailed features can be extracted from captured images, significantly reducing the time and errors associated with manual coding. Image recognition represents a key research area within computer vision, focusing on the classification and interpretation of visual content through neural networks, and has provided substantial support for fields such as medical image analysis.

In the present study, we objectively measured daily screen time duration in school-aged children using wearable cameras integrated with image recognition technology, and examined its association with obesity-related indicators, including both general and abdominal measures. We further investigated the combined effects of dietary intake, dietary behaviors, and physical activity on the relationship between daily screen time and obesity.

## 2. Materials and Methods

### 2.1. Study Design and Participants

This was a cross-sectional study conducted among grade 4 children in a primary school in Beijing from May to October 2017. This school was not a boarding school. Moreover, it did not specifically recruit students from ethnic minority groups or students with special skills. Children aged 9 to 10.9 years from the selected primary school were considered potential participants. Exclusion criteria included a history of severe cardiovascular, pulmonary, hepatic, or renal conditions (e.g., hypertension, tuberculosis); iatrogenic obesity; abnormal growth disorders (e.g., dwarfism, gigantism); physical disabilities; or any recent weight loss efforts (e.g., dieting, use of medications for weight reduction) within the last three months. This study was conducted in accordance with the Declaration of Helsinki and approved by the Institutional Review Board of Peking University Health Science Centre (ethics approval number: IRB00001052-17040). All participants and their parents provided informed consent and signed the consent forms.

Participants were recruited to achieve a 1:1 matched sample of children with obesity and normal-weight children. Matching was performed at the individual level by sex (one child with obesity matched to one child with normal weight of the same sex). The aim of the study was to evaluate the accuracy of traditional dietary recall method [17]. Therefore, the sample size was calculated based on the differences in daily energy intakes measured with and without the assistance of wearable cameras. The effect size (Cohen’s d) for the difference in energy intake between the two dietary recall methods was set at 0.4 [18]. Using a two-sided t-test with a significance level of 0.05, a sample size of 54 participants provided 80% power to detect this difference. For the current analysis, we further explored the associations between screen time, dietary behaviors, and obesity-related outcomes.

### 2.2. Assessment of Screen Time Exposure

During the study period, every participant wore a wearable camera (Narrative Clip 2, Taiwan, China) for one week. The camera was attached to their chest using a metal clip and elastic strap. In accordance with standard ethical procedures, participants were instructed to wear the camera when waking and remove it before going to bed. They were allowed to take off or turn off the camera if they felt uncomfortable. The camera was also removed during activities like bathing or swimming. It captured one photo approximately every 12 s. The ethical protocol of this study was developed based on the ethical framework for automated wearable cameras proposed by Kelly et al. [19]. The protocol for photo annotation has been described in detail in our previously published study [17].

Many image recognition methods achieved recognition ability by building neural networks, such as CNNs [20], and training these networks to extract features from images for classification purposes. Some methods also used clustering algorithms to classify different images from videos [21]. Additionally, techniques such as sliding window technology was used to capture texture information from different regions of an image, then obtaining classification results [22]. The recognition result can also be obtained by matching the similarity between the image predicted and the actual images in the knowledge base [23].

The study involved using a pre-trained ResNet model to detect if there was a screen in the images, then employing a large language model to generate textual descriptions. Then, the text description was matched with keywords and compared with prompts to obtain the final recognition result. Keyword matching was to determine whether the description contains keywords such as “Television”, “Phone”, “Computer”, or “Tablet”. “Projector” was also identified as a keyword during image recognition process. However, considering that students were routinely exposed to projectors in selected school (where projectors were used as instructional tools), the study focused primarily on screen use during children’s leisure time and did not take projector exposure into account. The first step in comparing prompts was to obtain prompts. Using a large language model, different categories of images in the training set were used to obtain descriptions as prompts. Then, the descriptions of the images predicted were matched with these prompts for similarity, and the classification was obtained based on the matching results.

### 2.3. Physical Activity Measurement

Free-living physical activity was objectively measured using ActiGraph GT3X accelerometer (Actigraph Corp, Pensacola, FL, USA) mounted on the right waist of each child by means of an elastic belt. Children were asked to wear the accelerometer for seven consecutive days, including 5 weekdays and 2 weekend days. According to the protocol, a 15 s sampling interval (‘epoch’) was to be used in physical activity data collection. Children were encouraged to wear the accelerometer from the moment they woke up in the morning until bedtime in the evening. Data from the accelerometers were analyzed using ActiLife v 6.8.2 software (ActiGraph Inc., Pensacola, FL, USA). Activity intensities were categorized based on count cut-off values: sedentary behavior activity (0–100 counts/min), light physical activity (101–2295 counts/min), moderate-intensity physical activity (2296–4011 counts/min), and vigorous physical activity (≥4012 counts/min) [24]. Moderate-to-vigorous physical activity (MVPA) time included the sum of time spent in moderate-intensity physical activity and vigorous physical activity. Accelerometer wear time was also obtained from the accelerometer. The MVPA proportion was defined as the ratio of MVPA time to the total physical activity time.

### 2.4. Dietary Intake and Behavior Measurement

The dietary intake factors included 24 h energy (kcal/day), protein (g/day), carbohydrates (g/day) and fat (g/day) intake based on a camera-assisted 3-day 24 h dietary recall. The study protocol and the feasibility of using wearable cameras to assess children’s dietary intake in China has been published in detail elsewhere [17]. For the extraction of dietary behavior information, researchers sequentially reviewed the wearable camera images to identify all relevant eating events on the dietary recall days. Key photos marking the start and end of each event were recorded using a data summary sheet. The average duration per meal was calculated by subtracting the timestamp of the starting photo from that of the ending photo for the same eating event, with the result expressed in minutes. The average eating speed per meal (g/min) was calculated by dividing the average edible weight of food consumed per meal obtained from the dietary recall by the average duration per meal. If any photo within a given eating event contained an operational screen device (television, phone, computer, or pad), the event was classified as a meal with screen. The proportion of meals with screen was calculated as the number of such events divided by the total number of recorded eating events. Similarly, if any photo within an eating event showed the presence of parents, grandparents, siblings, classmates, or friends, and the participant was observed to be eating or interacting with them, the event was classified as a meal with social interaction. The proportion of meals with social interaction was then calculated by dividing the number of such events by the total number of recorded eating events.

### 2.5. Outcome Measurement

Anthropometric measurements, including height, weight, waist circumference (WC), and hip circumference (HC) were collected. Children were asked to stand straight without shoes while wearing light weight clothing. Weight was measured using a lever-type scale with a precision of 0.1 kg, height was recorded with a stadiometer accurate to 0.1 cm, and WC was measured with a non-elastic tape to the nearest 0.1 cm. Each measurement was taken twice, and the average of the two was used for the final analysis. Body composition was measured using an eight-electrode bioelectrical impedance analysis device (TANITA MC-780MA, Tokyo, Japan). All measurements were conducted by trained personnel using standardized procedures. Before each use, the measurement devices were all calibrated according to standard operating procedure.

Body mass index (BMI) was calculated by dividing weight by the square of height. Age- and sex-specific BMI Z-scores were calculated using the BMI (kg/m^2^) standards defined by the WHO [25]. Weight-to-height ratio (WHtR) was computed by dividing waist circumference (WC) by height. WHtR > 0.50 has been widely considered an optimal cut-off for identifying central obesity and cardiometabolic risk in youth [26]. Age- and sex-specific WC ≥ 90th percentile was classified according to the high waist circumference criteria for school-aged children and adolescents in China [27]. Age- and sex-specific BF% ≥ 75th percentile was based on a set of body fat reference values for Chinese children and adolescents aged 3 to 18 years [28]. This study based on data from the China Child and Adolescent Cardiovascular Health study, which included fat measurements (including BF%) from a nationwide sample of 12,790 children and adolescents aged 3–18 years. The sex- and age-specific percentiles of body fat were estimated using the lambda-mu-sigma method.

Children with obesity as defined by the ‘Screening for overweight and obesity among school-age children and adolescents in China’ were eligible for the case group, while children classified as normal weight were included in the control group [29]. Children identified as underweight or overweight did not meet the inclusion criteria.

### 2.6. Covariates

Potential confounders including sociodemographic variables (age, sex, birth weight and socioeconomic status) and behavior factors (dietary intake, total physical activity duration in minutes/day, and sleep duration). Information on the child’s birth status, sleep habits, parental date of birth, height, weight, educational level, and occupation were collected via a parent-completed questionnaire. This questionnaire was distributed to students to take home and was completed by the parent who was primarily responsible for the child’s daily care and diet. It was then returned to the school and collected by research staff the following day. Parental BMI was calculated as weight in kilograms divided by the square of height in meters (kg/m^2^) based on self-reported height and weight from questionnaire. Socioeconomic status was assessed using the Green’s score [30], with a higher score reflecting a higher socioeconomic status. Green’s score = 0.5 × (father’s level of education score × 0.7 + father’s occupation score × 0.4 + mother’s level of education score × 0.7 + mother’s occupation score × 0.4).

### 2.7. Statistical Analysis

Descriptive statistics were used to summarize the sociodemographic characteristics of the children, anthropometric measurements and screen time. Quantitative variables were reported as means with standard deviations, and qualitative variables were analyzed as counts and percentages. Continuous variables were performed between groups using independent-samples t-tests, and categorical variables were compared using the chi-square test or Fisher’s exact test as appropriate. Logistic regression models were applied to calculate adjusted odds ratios (ORs) and 95% confidence intervals (CIs) for the associations between screen time and indicators of general and abdominal adiposity. The median screen time (1.3 h) of children with normal weight was used as the cutoff. Similarly, the median protein intake proportion (17.6%), proportion of meals with screen (20.0%), proportion of meals with social interaction (96%), MVPA proportion (16%) of children with normal weight were used as the cutoff. With respect to the time spent on television, phone, computer, and tablet, time of children with normal weight was also used as the cutoff. GAMs (Generalized Additive Models) were used to calculate the nonlinear relationships between total screen time and obesity indicators. All models adjusted for sociodemographic characteristics (sex and Green’s score) and behavior factors (physical activity time and dietary intake). In addition, we ran a post hoc power analysis using the observed effect sizes to help in interpreting the main results. Statistical significance was set at a *p*-value of <0.05. All statistical analyses were conducted with R version 4.1.0 (R Development Core Team, Vienna, Austria).

## 3. Results

A total of 62 fourth-grade primary school students met the eligibility criteria based on the aforementioned standards. Following the exclusion of 10 participants who failed to complete the 3-day dietary recall, 52 students were ultimately enrolled in the study. Characteristics of the participants were given in Table 1. Age and birth weight were similar among the children with obesity and the normal weight children. However, children with obesity were more likely to have a mother with higher BMI (25.68 ± 3.51 vs. 22.32 ± 2.40). There were no differences among dietary intake (including energy, protein, carbohydrates and fat intake), total physical activity level (for a whole week), or sleep duration between children with normal weight and those with obesity. MVPA time more than 1 h/day was rare in children, with only 8% of participants meeting this recommendation. Children with normal weight reported a mean of 83.15 ± 36.86 min weekly screen time, while the ones with obesity reported a mean of 94.91 ± 35.44 min/week. Children with obesity were found to spend more time watching television compared to those with normal weight (19.66 ± 19.80 vs. 10.73 ± 9.76, *p*-value < 0.05). During the whole week, children with obesity having more screen time compared to normal weight children, but the result was not significant (Table 1).

Children were also categorized into less screen time and more screen time groups, respectively. Results showed that children with longer screen time consumed more carbohydrates (288.06 ± 72.77 vs. 246.07 ± 72.44 g). They also had a longer average duration per meal (14.84 ± 4.83 vs. 11.14 ± 3.31 min) and a higher proportion of meals with screen exposure (0.28 ± 0.24 vs. 0.14 ± 0.17). In terms of sedentary behavior, children with more screen time spent more time in sedentary behavior (480.08 ± 54.87 vs. 429.80 ± 71.06 min/day), compared to those with less screen time. In terms of physical activity, children with more screen time had a lower proportion of moderate-to-vigorous physical activity time (0.18 ± 0.04 vs. 0.14 ± 0.03) compared to those with less screen time (Supplementary Table S1). We also conducted additional analyses by categorizing children’s use of four screen device types (television, phone, computer, and tablet) into more-use and less-use groups. The results indicated that children with more television use had longer average meal duration and a higher proportion of meals involving screen use. Among those with higher phone use, the proportion of meals with screen exposure was also more. Children who used computers exhibited a greater proportion of social interaction during meals, longer sedentary behavior duration, and higher levels of MVPA. For children with higher tablet, we observed increased carbohydrate intake, longer average meal durations, slower eating speeds, and more sedentary behavior time (Supplementary Tables S2–S5).

**Figure 1:**
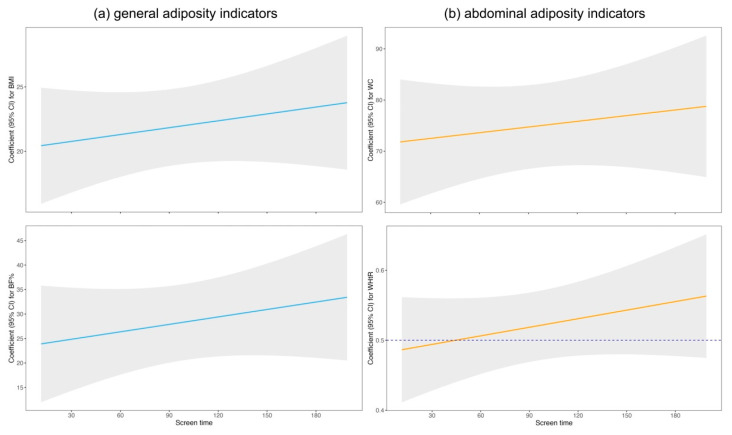
Generalized additive models for screen time and adiposity indicators. (**a**) screen time and general adiposity indicators; (**b**) screen time and abdominal adiposity indicators. Models were adjusted for Green’s score, MVPA time and total energy intake. Gray area stands for 95% CI bands. Dotted blue lines in WHtR represents 0.50.

Odds ratios for the association between screen time ≥ 1.3 h and adiposity indicators (both general and abdominal) are shown in Table 2. We observed a potential positive association between time spent on screen for the whole week and obesity in model 2 and 3 (Model 2: OR = 3.62, 95% CI = 1.02, 12.93, *p*-value = 0.047; Model 3: OR = 4.25, 95% CI = 1.09, 16.53, *p*-value = 0.037). Results further suggested a possible positive correlation between screen time duration and BF% ≥ 75th in all models (Model 1: OR = 3.98, 95% CI: 1.09, 14.58, *p*-value = 0.037; Model 2: OR = 4.54, 95% CI: 1.09, 18.86, *p*-value = 0.037; Model 3: OR = 6.14, 95% CI: 1.29, 29.10, *p*-value = 0.022). However, no significant results were observed across all models among other obesity related indicators (WHtR ≥ 0.50, WC ≥ 90th). Given the relatively small sample size, the wide confidence intervals suggested that these results should be interpreted with caution. In the GAM models, similar linear trends were observed in the relationship between screen time and all adiposity indicators (BMI, WC, BF% and WHtR) (Figure 1).

The association between cross variable (combining screen time and dietary intake, dietary behavior, or MVPA time) and obesity was presented in Table 3. More screen time combined with lower protein intake was associated with increased odds of childhood obesity (unadjusted: OR = 7.43, 95% CI: 1.23, 45.01, *p*-value = 0.029; adjusted: OR = 9.57, 95% CI: 1.31, 70.14, *p*-value = 0.026). Additionally, children with longer screen time and a higher proportion of meals with screen exposure appeared to have increased odds of obesity (unadjusted: OR = 6.00, 95% CI: 1.26, 28.55, *p*-value = 0.024; adjusted: OR = 6.40, 95% CI: 1.22, 33.61, *p*-value = 0.028). Similarly, longer screen time combined with a lower proportion of meals with social interaction was also associated with an increased odds of obesity (unadjusted: OR = 6.00, 95% CI: 1.08, 33.38, *p*-value = 0.041; adjusted: OR = 5.90, 95% CI: 1.01, 34.59, *p*-value = 0.049). There was a potential association between prolonged screen time and reduced MVPA time with increased odds of obesity. The ORs were significant for the more screen time (≥1.3 h) and less MVPA proportion (<16%) group (unadjusted: OR = 4.44, 95% CI: 1.08, 18.36, *p*-value = 0.039; adjusted: OR = 5.21, 95% CI: 1.11, 24.43, *p*-value = 0.037). However, insignificant interactions were found between more screen time and less MVPA proportion (relative excess risk due to interaction (RERI): 0.72, 95% CI = −7.37, 8.80; attributable proportion due to interaction (AP): 0.14, 95% CI: −1.36, 1.64; synergy index (SI): 1.21, 95% CI: 0.13, 11.2), as well as less protein intake, more proportion of meals with screen, less proportion of meals with social interaction, and less MVPA time.

## 4. Discussion

This study investigated the association between screen time and obesity-related indicators (including both general and abdominal obesity) in school-aged children. The findings revealed the difference in screen time between children with and without obesity, where children with obesity tended to spend more time on screens. Compared with children with lower screen time exposure, those with higher exposure tended to consume more carbohydrates, also had a longer average duration per meal, a higher proportion of meals with screen exposure, spent more time in sedentary behavior, and had a lower proportion of time spent in MVPA. Longer screen time was positively associated with an increased risk of obesity, the same after adjusting for other confounding factors, including sociodemographic variables, dietary intake and physical activity. We discovered that longer screen time duration combined with less MVPA proportion positively correlated with higher risk of obesity compared with less screen time and more MVPA proportion among these children. This adverse association was also observed when longer screen time duration was combined with lower protein intake, a higher proportion of meals with screen exposure, and a lower proportion of meals with social interaction. These findings may provide evidence for parents, families and pedagogues in setting and communicating screen time limits for this age group. In addition, the findings may inform the development of interventions promoting physical activity in primary school children. Additionally, it is important to increase the proportion of protein intake in children’s daily diets. Parents should also focus on fostering healthy eating habits in children by avoiding screen use during meals, and by engaging in shared mealtimes with their children.

The findings of this study reinforce the conclusion that increased screen time raises the risk of obesity in children and adolescents, consistent with previous research outcomes. A cross-sectional study using data from the 2016–2017 National Survey of Children’s Health in the United States found that for adolescents who did not meet physical activity guidelines, engaging in TV watching or video gaming for ≥1 h per day was associated with higher BMI [31]. A systematic review found that compared to screen time < 2 h/day, children under 18-year-old with screen time ≥ 2 h/day were associated with an increased risk of overweight or obesity (OR = 1.67, 95% CI: 1.48, 1.88) [12]. Another systematic review assessing the relationship between screen time and the likelihood of being overweight/obesity in adolescents, included studies involving 10- to 20-year-olds, and found that adolescents with the highest screen time had a 1.27-fold increased risk of overweight or obesity [32]. However, these studies did not find evidence of a nonlinear association between increased screen time and overweight/obesity. The difficulty in exploring such nonlinear associations may come from most screen time measurements relying on self-reported questionnaires, which can introduce bias. Our study objectively measured children’s screen exposure time using wearable cameras and used GAM to explore possible nonlinear relationships between screen time and obesity-related indicators, identifying linear trends were observed between screen time and all adiposity indicators. This study also included central obesity indicators (WHtR, WC) and BF% as outcome measures, positive correlation was found between screen time and BF%. Similarly, a systematic review examining the relationship between central obesity and screen time in children and adolescents also found no significant association between screen time and the likelihood of central obesity [33].

There are several mechanisms that could explain the relationship between screen time and obesity. It could be the result of a direct effect on obesity, or indirectly through pathways such as reduced physical activity, decreased sleep duration, and other related factors. Excessive screen time is considered as sedentary behavior, which have had a well-established link to childhood and adolescent obesity, with WHO having set specific time limits [5]. Screen time also contributed to obesity through various indirect pathways. Previous studies have shown that screen behavior may influence dietary habits, increasing children’s caloric intake. Consistent with previous studies, our study also found that children with longer screen time tended to consume more energy and carbohydrates. Children may be more prone to overeating without being hungry when they were distracted in front of screens [34], especially for snacks and SSBs (sugar-sweetened beverages), which can lead to a decreased consumption of healthy foods like fruits and vegetables during meals [35]. In our study, longer screen time was associated with longer meal durations and a higher proportion of meals consumed while watching screens. At the same time, screen time can lead to energy expenditure decrease by increasing sedentary behavior and reducing physical activity time. In this study we controlled for dietary intake and physical activity time in the model, and screen time remained associated with obesity and obesity related indicators. This significant association remained evident even after simultaneously accounting for dietary intake, dietary behaviors, and physical activity. However, due to the relatively small sample size, the confidence intervals of the ORs were wide; thus, these findings should be interpreted with caution and warrant information in studies with larger samples.

Increased screen time in children may facilitate the development of obesity by reducing physical activity time and increasing sedentary behavior. We found that higher proportion of screen time combined with less MVPA proportion (as a percentage of total physical activity time) was significantly associated with a higher risk of obesity, compared to children with less screen time and more MVPA. Other groups, such as those with only less MVPA (<16%) and less screen time (<1.3 h/day), also showed harmful effects on obesity, but the results were not significant. MVPA was associated with numerous health benefits and played a critical role in the prevention and treatment of childhood and adolescent obesity [36]. Children with obesity generally engaged in less MVPA, and this study also found that although children with obesity had more total physical activity time on average, the proportion of MVPA was less. Increased MVPA may have a counteracting effect on the harmful role of screen time duration. Therefore, it is important to promote an increase in MVPA among school-aged children at an early stage, with a particular focus on increasing the proportion of MVPA within their overall physical activity.

This study had several strengths. First, when using wearable cameras to objectively record children’s screen exposure times and types, there is a benefit over self-reported data which is prone to suffer from recall and reporting bias and thus hamper reflecting the real time exposure. Additionally, using image recognition technology to analyze the photos collected by the wearable cameras, significantly reduced labor and time costs for the participants. Second, this study took both general and abdominal obesity indicators as obesity related outcomes, whereas some studies rely only on BMI as an obesity related outcome [31]. Furthermore, the innovative use of the GAM allowed us to explore potential nonlinear associations between screen time and childhood obesity, helping to identify possible threshold points.

This study had limitations as well. First, while the image recognition technology used in this study demonstrated good recognition capabilities, there are still limitations in differentiating the specific activities (e.g., playing video games or using social media). However, by applying an innovative and objective method to measure children’s screen exposure, this study provides important insights and demonstrates the feasibility of using wearable cameras in pediatric obesity research. Second, the relatively small sample size of this study resulted in wide confidence intervals for some of the associations. Post hoc power analysis indicated that the statistical power of this study was 70.7%, which falls below the conventional threshold of 80%. Thus, the findings should be interpreted with caution and require validation in larger and more diverse populations.

## 5. Conclusions

We objectively collected screen behavior and obesity-related outcomes in Chinese school-age children using wearable cameras, revealing a positive correlation between screen time and obesity. More screen time may be associated with higher energy and carbohydrate intake, longer meal duration, a higher proportion of meals consumed with screens, and increased sedentary behavior and lower MVPA level. Additionally, children with more screen time and less MVPA proportion, less protein intake, more proportion of meals with screen, or less proportion of meals with social interaction had higher risk of obesity. These findings suggested that future obesity interventions should consider limiting children’s screen time while promoting higher proportions of MVPA in their overall physical activity and encouraging greater dietary protein intake. Additionally, we highlighted the importance of cultivating healthy eating behaviors by reducing screen use during meals and promoting shared family mealtimes to support childhood obesity prevention and management.

## Figures and Tables

**Table 1:** Characteristics of the participants.

Variables	Children with Normal Weight (N = 26)	Children with Obesity (N = 26)	95% CI for Mean Difference	*p*-Value
	Mean ± SD or N (%)	Mean ± SD or N (%)		
Age (years)	9.78 ± 0.44	9.75 ± 0.45	−0.21, 0.28	0.774
Gender (%)			-	-
Male	13 (50)	13 (50)		
Female	13 (50)	13 (50)		
Socioeconomic status				
Green’s score	55.28 ± 6.50	53.67 ± 7.33	−2.30, 5.51	0.413
Birth weight (kg)	3.34 ± 0.37	3.49 ± 0.38	−0.36, 0.06	0.151
Mother’s BMI (kg/m^2^)	22.32 ± 2.40	25.68 ± 3.51	−5.04, −1.68	**<0.001**
Children’s BMI (kg/m^2^)	16.47 ± 1.49	25.11 ± 1.95	−9.62, −7.68	**<0.001**
Weight (cm)	32.14 ± 4.01	51.31 ± 6.23	−22.10, −16.24	**<0.001**
Height (cm)	139.53 ± 4.80	142.75 ± 5.52	−6.10, −0.33	**0.030**
Waist circumference (cm)	60.76 ± 5.26	82.71 ± 6.11	−25.13, −18.78	**<0.001**
Hip circumference (cm)	72.13 ± 4.67	88.77 ± 4.50	−19.19, −14.08	**<0.001**
WHtR	0.44 ± 0.03	0.58 ± 0.03	−0.16, −0.13	**<0.001**
BF %	15.99 ± 6.51	36.14 ± 3.78	−23.13, −17.16	**<0.001**
Dietary intake				
Energy (kcal)	1965.85 ± 606.09	1942.33 ± 452.95	−275.12, 322.17	0.875
Protein (g)	89.62 ± 40.79	77.70 ± 21.03	−6.31, 30.15	0.194
Protein intake proportion (%)			-	0.258
<17.6%	13 (50)	18 (69)		
≥17.6%	13 (50)	8 (31)		
Carbohydrates (g)	266.40 ± 82.28	275.80 ± 67.98	−51.47, 32.68	0.656
Fat (g)	63.43 ± 23.40	61.85 ± 25.77	−12.13, 15.30	0.818
Dietary behavior				
Average duration per meal (min)	13.01 ± 3.85	13.68 ± 5.35	−3.27, 1.94	0.609
Average edible weight of food consumed per meal (g)	354.10 ± 113.26	354.97 ± 82.74	−56.24, 54.51	0.975
Average eating speed per meal (g/min)	28.66 ± 9.98	28.96 ± 11.80	−6.39, 5.79	0.920
Proportion of meals with screen (%)	0.20 ± 0.22	0.25 ± 0.22	−0.18, 0.07	0.404
Proportion of meals with social interaction (%)	0.89 ± 0.13	0.79 ± 0.18	0.02, 0.19	**0.022**
Screen behavior				
Screen time (min/day)	83.15 ± 36.86	94.91 ± 35.44	−31.90, 8.38	0.247
Screen time (%)			-	0.090
<1.3 h	14 (67)	7 (33)		
≥1.3 h	12 (39)	19 (61)		
Time spent on television (min/day)	10.73 ± 9.76	19.66 ± 19.78	−17.70, −0.16	**0.046**
Time spent on phone (min/day)	5.65 ± 5.18	5.64 ± 7.13	−3.47, 3.49	0.997
Time spent on computer (min/day)	66.0 ± 32.2	69.0 ± 27.7	−19.69, 13.82	0.727
Time spent on tablet (min/day)	0.73 ± 0.58	0.64 ± 0.44	−0.19, 0.38	0.512
Physical activity				
Physical activity time (min/day)	242.59 ± 51.38	264.17 ± 53.25	−50.73, 7.56	0.143
MVPA time ≥ 1 h/day (%)			-	-
No	24 (50)	24 (50)		
Yes	2 (50)	2 (50)		
MVPA time proportion (%)	0.16 ± 0.04	0.15 ± 0.04	−0.01, 0.04	0.207
MVPA time proportion (%)			-	0.154
<16%	13 (41)	19 (59)		
≥16%	13 (65)	7 (35)		

Abbreviation: SD: standard error; CI: confidence interval; BMI: body mass index; WHtR: waist-to-height ratio; BF %: percentage of body fat; MVPA: moderate-to-vigorous physical activity. Bold indicates statistically significant at the *p*-value < 0.05 level.

**Table 2:** Association of general and abdominal adiposity indicators and more screen time.

Model ^a^	Obesity			WHtR ≥ 0.50			WC ≥ 90th			BF% ≥ 75th		
	OR	95% CI	*p*-Value	OR	95% CI	*p*-Value	OR	95% CI	*p*-Value	OR	95% CI	*p*-Value
1	3.17	0.99, 10.10	0.051	2.57	0.82, 8.04	0.104	2.11	0.68, 6.51	0.194	3.98	1.09, 14.58	**0.037**
2	3.62	1.02, 12.93	**0.047**	2.75	0.81, 9.40	0.106	2.28	0.68, 7.66	0.181	4.54	1.09, 18.86	**0.037**
3	4.25	1.09, 16.53	**0.037**	3.26	0.88, 12.08	0.077	2.52	0.71, 9.00	0.154	6.14	1.29, 29.10	**0.022**

^a^ More screen time: daily screen time ≥ 1.3 h. Model 1 was unadjusted; Model 2 adjusted for sex, Green’s score and total energy (kcal); Model 3 also adjusted for MVPA time. Abbreviation: WHtR: waist-to-height ratio; WC: waist circumference; BF %: percentage of body fat; OR: odd risk; CI: confidence interval. Bold indicates statistically significant at the *p*-value < 0.05 level.

**Table 3:** Association of obesity and cross variable combining screen time and dietary or physical activity factors.

Variable	Unadjusted Model			Adjusted Model ^b^		
	OR	95% CI	*p*-Value	OR	95% CI	*p*-Value
More protein intake ^a^	0.44	0.14, 1.38	0.161	0.42	0.13, 1.39	0.155
More proportion of meals with screen	2.58	0.84, 7.91	0.098	2.55	0.80, 8.15	0.114
Less proportion of meals with social interaction	2.71	0.85, 8.64	0.091	2.73	0.82, 9.08	0.102
More MVPA time	0.79	0.21, 3.02	0.734	0.63	0.14, 2.89	0.549
Less MVPA proportion	2.71	0.85, 8.64	0.091	2.94	0.84, 10.30	0.091
Screen time and protein intake proportion						
Less screen time + More protein intake	Ref.			Ref.		
Less screen time + Less protein intake	3.33	0.47, 23.47	0.227	4.29	0.56, 33.06	0.163
More screen time + More protein intake	4.80	0.68, 33.80	0.115	5.76	0.75, 44.04	0.091
More screen time + Less protein intake	7.43	1.23, 45.01	**0.029**	9.57	1.31, 70.14	**0.026**
Screen time and proportion of meals with screen						
Less screen time + Less proportion of meals with screen	Ref.			Ref.		
Less screen time + More proportion of meals with screen	1.87	0.28 12.46	0.515	2.01	0.29, 13.83	0.477
More screen time + Less proportion of meals with screen	2.50	0.52, 11.93	0.251	2.69	0.53, 13.61	0.231
More screen time + More proportion of meals with screen	6.00	1.26, 28.55	**0.024**	6.40	1.22, 33.61	**0.028**
Screen time and proportion of meals with social interaction						
Less screen time + More proportion of meals with social interaction	Ref.			Ref.		
Less screen time + Less proportion of meals with social interaction	1.00	0.16, 6.26	1.000	0.93	0.14, 6.20	0.937
More screen time + More proportion of meals with social interaction	1.14	0.18, 7.28	0.888	1.12	0.17, 7.42	0.906
More screen time + Less proportion of meals with social interaction	6.00	1.08, 33.38	**0.041**	5.90	1.01, 34.59	**0.049**
Screen time and MVPA time						
Less screen time + More MVPA time	Ref.			Ref.		
Less screen time + Less MVPA time	0.00	0.00, Inf	-	0.00	0.00, Inf	-
More screen time + More MVPA time	2.48	0.70, 8.74	0.159	2.77	0.70 10.95	0.147
More screen time + Less MVPA time	3.43	0.65, 18.22	0.148	4.19	0.66 26.40	0.127
Screen time and MVPA proportion						
Less screen time + More MVPA proportion	Ref.			Ref.		
Less screen time + Less MVPA proportion	1.87	0.28, 12.46	0.515	2.18	0.30, 15.56	0.439
More screen time + More MVPA proportion	2.50	0.35, 18.04	0.364	3.31	0.38, 29.15	0.281
More screen time + Less MVPA proportion	4.44	1.08, 18.36	**0.039**	5.21	1.11, 24.43	**0.037**

Abbreviation: OR: odd risk; CI: confidence interval; MVPA: moderate-to-vigorous physical activity. ^a^ Less protein intake: protein intake < 17.6%; More protein intake: protein intake ≥ 17.6%; Less screen time: daily screen time < 1.3 h; More screen time: daily screen time ≥ 1.3 h; Less proportion of meals with screen: proportion < 20.0%; More proportion of meals with screen: proportion ≥ 20.0%; Less proportion of meals with social interaction: proportion < 96%; More proportion of meals with social interaction: proportion ≥ 96%; Less MVPA time: MVPA time < 30 min; More MVPA time: MVPA time ≥ 30 min; Less MVPA proportion: MVPA proportion < 16%; More MVPA proportion: MVPA proportion ≥ 16%. ^b^ Model for dietary intake was adjusted for sex, Green’s score and MVPA time; Model for physical activity was adjusted for sex, Green’s score and total energy (kcal). Bold indicates statistically significant at the *p*-value < 0.05 level. Ref. indicates the reference group.

## Data Availability

The original contributions presented in this study are included in the article. Further inquiries can be directed to the corresponding author.

## Supplementary Materials

The following supporting information can be downloaded at: https://www.mdpi.com/article/10.3390/nu17182990/s1. Table S1. Comparison of diet and physical activity by screen time levels; Table S2. Comparison of diet and physical activity by television time levels; Table S3. Comparison of diet and physical activity by phone time levels; Table S4. Comparison of diet and physical activity by computer time levels; Table S5. Comparison of diet and physical activity by tablet time levels.

## Abbreviations

The following abbreviations are used in this manuscript:

BF%	Percentage of body fat
BMI	Body mass index
CI	Confidence interval
CNN	Convolutional neural network
GAM	Generalized additive model
HC	Hip circumference
MVPA	Moderate-to-vigorous physical activity
OR	Odds ratio
ResNet	Residual network
SD	standard error
WC	Waist circumference
WHO	World health organization
WHtR	Weight-to-height ratio

## References

[1] Caprio S., Santoro N., Weiss R. (2020). Childhood obesity and the associated rise in cardiometabolic complications. Nat. Metab..

[2] Deal B.J., Huffman M.D., Binns H., Stone N.J. (2020). Perspective: Childhood Obesity Requires New Strategies for Prevention. Adv. Nutr..

[3] Chaput J.P., Janssen I., Spence J.C. (2012). Time spent sedentary and active and cardiometabolic risk factors in children. JAMA.

[4] World Health Organization Guidelines on Physical Activity, Sedentary Behaviour and Sleep for Children Under 5 Years of Age. https://apps.who.int/iris/handle/10665/311664.

[5] World Health Organization WHO Guidelines on Physical Activity and Sedentary Behaviour. https://www.who.int/publications/i/item/9789240015128.

[6] Nagata J.M., Ganson K.T., Iyer P., Chu J., Baker F.C., Pettee Gabriel K., Garber A.K., Murray S.B., Bibbins-Domingo K. (2022). Sociodemographic Correlates of Contemporary Screen Time Use among 9- and 10-Year-Old Children. J. Pediatr..

[7] Song Y., Li L., Xu Y., Pan G., Tao F., Ren L. (2020). Associations between screen time, negative life events, and emotional and behavioral problems among Chinese children and adolescents. J. Affect. Disord..

[8] Sina E., Buck C., Veidebaum T., Siani A., Reisch L., Pohlabeln H., Pala V., Moreno L.A., Molnar D., Lissner L. (2021). Media use trajectories and risk of metabolic syndrome in European children and adolescents: The IDEFICS/I.Family cohort. Int. J. Behav. Nutr. Phys. Act..

[9] Shqair A.Q., Pauli L.A., Costa V.P.P., Cenci M., Goettems M.L. (2019). Screen time, dietary patterns and intake of potentially cariogenic food in children: A systematic review. J. Dent..

[10] Tooth L.R., Moss K.M., Mishra G.D. (2021). Screen time and child behaviour and health-related quality of life: Effect of family context. Prev. Med..

[11] Brushe M.E., Haag D.G., Melhuish E.C., Reilly S., Gregory T. (2024). Screen Time and Parent-Child Talk When Children Are Aged 12 to 36 Months. JAMA Pediatr..

[12] Fang K.A.-O., Mu M., Liu K., He Y. (2019). Screen time and childhood overweight/obesity: A systematic review and meta-analysis. Child Care Health Dev..

[13] Saunders T.J., Vallance J.K. (2017). Screen Time and Health Indicators Among Children and Youth: Current Evidence, Limitations and Future Directions. Appl. Health Econ. Health Policy.

[14] Cardoso-Leite P., Buchard A., Tissieres I., Mussack D., Bavelier D. (2021). Media use, attention, mental health and academic performance among 8 to 12 year old children. PLoS ONE.

[15] Lowe B.M., Smith M., Jaine R., Stanley J., Gage R., Signal L. (2023). Watching the watchers: Assessing the nature and extent of children’s screen time using wearable cameras. N. Z. Med. J..

[16] Thomas G.A.-O., Bennie J.A.-O., De Cocker K.A.-O., Dwi Andriyani F.A.-O., Booker B.A.-O., Biddle S.A.-O. (2022). Using Wearable Cameras to Categorize the Type and Context of Screen-Based Behaviors Among Adolescents: Observational Study. JMIR Pediatr. Parent..

[17] Zhou Q., Wang D., Mhurchu C.N., Gurrin C., Zhou J., Cheng Y., Wang H. (2019). The use of wearable cameras in assessing children’s dietary intake and behaviours in China. Appetite.

[18] Gemming L., Rush E., Maddison R., Doherty A., Gant N., Utter J., Ni Mhurchu C. (2015). Wearable cameras can reduce dietary under-reporting: Doubly labelled water validation of a camera-assisted 24 h recall. Br. J. Nutr..

[19] Kelly P., Marshall S.J., Badland H., Kerr J., Oliver M., Doherty A.R., Foster C. (2013). An ethical framework for automated, wearable cameras in health behavior research. Am. J. Prev. Med..

[20] Zhang X.-J., Lu Y.-F., Zhang S.-H. (2016). Multi-task learning for food identification and analysis with deep convolutional neural networks. J. Comput. Sci. Technol..

[21] Pan X., He J., Zhu F. Muti-stage hierarchical food classification. Proceedings of the 8th International Workshop on Multimedia Assisted Dietary Management.

[22] Qiu J., Lo F.P.-W., Sun Y., Wang S., Lo B. (2022). Mining discriminative food regions for accurate food recognition. arXiv.

[23] Shao Z., He J., Yu Y.-Y., Lin L., Cowan A., Eicher-Miller H., Zhu F. (2022). Towards the creation of a nutrition and food group based image database. arXiv.

[24] Evenson K.R., Catellier D.J., Gill K., Ondrak K.S., McMurray R.G. (2008). Calibration of two objective measures of physical activity for children. J. Sports Sci..

[25] World Health Organization Growth Reference Data for 5–19 Years. https://www.who.int/tools/growth-reference-data-for-5to19-years.

[26] Lo K., Wong M., Khalechelvam P., Tam W. (2016). Waist-to-height ratio, body mass index and waist circumference for screening paediatric cardi--metabolic risk factors: A meta-analysis. Obes. Rev..

[27] (2018). High Waist Circumference Screening Threshold Among Children and Adolescents Aged 7~18 Years.

[28] Dong H., Yan Y., Liu J., Cheng H., Zhao X., Shan X., Huang G., Mi J., Mi J., Liu J. (2021). Reference centiles for evaluating total body fat development and fat distribution by dual-energy x-ray absorptiometry among children and adolescents aged 3–18 years. Clin. Nutr..

[29] (2018). Screening for Overweight and Obesity Among School-Age Children and Adolescents.

[30] Zhai Y., Sulayiman X., Li W., Shen C., Zhao W., Shi X. (2013). The relationship between socioeconomic status and overweight and obesity among elementary school children in China. Zhonghua Yu Fang Yi Xue Za Zhi [Chin. J. Prev. Med.].

[31] Bakour C., Mansuri F., Johns-Rejano C., Crozier M., Wilson R., Sappenfield W. (2022). Association between screen time and obesity in US adolescents: A cross-sectional analysis using National Survey of Children’s Health 2016–2017. PLoS ONE.

[32] Haghjoo P., Siri G., Soleimani E., Farhangi M.A., Alesaeidi S. (2022). Screen time increases overweight and obesity risk among adolescents: A systematic review and dose-response meta-analysis. BMC Prim. Care.

[33] Ghasemirad M., Ketabi L., Fayyazishishavan E., Hojati A., Maleki Z.H., Gerami M.H., Moradzadeh M., Fernandez J.H.O., Akhavan-Sigari R. (2023). The association between screen use and central obesity among children and adolescents: A systematic review and meta-analysis. J. Health Popul. Nutr..

[34] Nagata J.A.-O., Iyer P., Chu J., Baker F.C., Pettee Gabriel K., Garber A.K., Murray S.A.-O., Bibbins-Domingo K., Ganson K.A.-O. (2021). Contemporary screen time modalities among children 9–10 years old and binge-eating disorder at one-year follow-up: A prospective cohort study. Int. J. Eat. Disord..

[35] Huo J., Kuang X., Xi Y., Xiang C., Yong C., Liang J., Zou H., Lin Q.A.-O. (2022). Screen Time and Its Association with Vegetables, Fruits, Snacks and Sugary Sweetened Beverages Intake among Chinese Preschool Children in Changsha, Hunan Province: A Cross-Sectional Study. Nutrients.

[36] Janssen X., Basterfield L., Parkinson K.N., Pearce M.S., Reilly J.K., Adamson A.J., Reilly J.J. (2019). Non-linear longitudinal associations between moderate-to-vigorous physical activity and adiposity across the adiposity distribution during childhood and adolescence: Gateshead Millennium Study. Int. J. Obes..

