# Supplementary material for: Wearable Camera-Based Objective Screen Time and Its Combined Associations with Dietary and Physical Activity Factors in Relation to Childhood Obesity
Source: Nutrients. 2025 Sep 18;17(18):2990. doi: 10.3390/nu17182990 (PMC12472260; doi:10.3390/nu17182990)
Supplement: Supplementary file 1 [file nutrients-17-02990-s001.zip › nutrients-3844802-supplementary.pdf]

Supplement Table S1. Comparison of diet and physical activity by screen time levels.

Variables	Less screen time [a] (N = 21)	More screen time (N = 31)	*p*-value
	**Mean ± SD or N (%)**	**Mean ± SD or N (%)**	
Dietary intake			
Energy (kcal)	1794.57 ± 556.90	2062.15 ± 490.61	0.082
Protein (g)	80.58 ± 36.65	85.75 ± 30.18	0.596
Protein intake proportion (%)			0.557
< 17.6%	11 (52)	20 (65)	
≥ 17.6%	10 (48)	11 (35)	
Carbohydrates (g)	246.07 ± 72.44	288.06 ± 72.77	**0.047**
Fat (g)	57.09 ± 23.54	66.40 ± 24.60	0.177
Dietary behavior			
Average duration per meal (min)	11.14 ± 3.31	14.84 ± 4.83	**0.002**
Average edible weight of food consumed per meal (g)	339.07 ± 112.32	365.01 ± 87.77	0.379
Average eating speed per meal (g/min)	32.86 ± 14.01	26.07 ± 6.99	**0.050**
Proportion of meals with screen (%)	0.14 ± 0.17	0.28 ± 0.24	**0.019**
Proportion of meals with social interaction (%)	0.82 ± 0.19	0.86 ± 0.15	0.400
Sedentary behavior			
Sedentary behavior (min/day)	429.80 ± 71.06	480.08 ± 54.87	**0.010**
Physical activity			
Physical activity time (min/day)	245.85 ± 50.32	258.48 ± 54.86	0.396
MVPA time (min/day)	44.03 ± 15.10	37.25 ± 12.84	0.100
MVPA time ≥ 1h/day (%)			0.291
No	18 (86)	30 (97)	
Yes	3 (14)	1 (3)	
MVPA time proportion (%)	0.18 ± 0.04	0.14 ± 0.03	**0.002**
MVPA time proportion (%)			**0.002**
< 16%	14 (67)	6 (19)	
≥ 16%	7 (33)	25 (81)	

[a] Median time of children with normal weight spent on screen time was used as the cutoff. Abbreviation: SD: standard error; MVPA: moderate-to-vigorous physical activity. Bold indicates statistically significant at the *p*-value < 0.05 level.

Supplement Table S2. Comparison of diet and physical activity by television time levels.

Variables	Less television time [a] (N = 21)	More television time (N = 31)	*p*-value
	**Mean ± SD or N (%)**	**Mean ± SD or N (%)**	
Dietary intake			
Energy (kcal)	1798.60 ± 379.44	2059.42 ± 594.11	0.059
Protein (g)	74.41 ± 26.94	89.93 ± 35.11	0.078
Protein intake proportion (%)			0.572
< 17.6%	14 (67)	17 (55)	
≥ 17.6%	7 (33)	14 (45)	
Carbohydrates (g)	255.91 ± 53.97	281.39 ± 85.54	0.194
Fat (g)	56.02 ± 18.25	67.12 ± 27.15	0.084
Dietary behavior			
Average duration per meal (min)	10.97 ± 2.89	14.95 ± 4.91	**< 0.001**
Average edible weight of food consumed per meal (g)	333.32 ± 98.66	368.90 ± 96.83	0.206
Average eating speed per meal (g/min)	32.62 ± 13.79	26.23 ± 7.43	0.062
Proportion of meals with screen (%)	0.13 ± 0.17	0.29 ± 0.23	**0.005**
Proportion of meals with social interaction (%)	0.84 ± 0.18	0.84 ± 0.16	0.885
Sedentary behavior			
Sedentary behavior (min/day)	449.85 ± 74.23	466.50 ± 60.36	0.398
Physical activity			
Physical activity time (min/day)	255.41 ± 50.08	252.01 ± 55.57	0.820
MVPA time (min/day)	41.75 ± 12.67	38.80 ± 15.01	0.449
MVPA time ≥ 1h/day (%)			1.000
No	19 (90)	29 (94)	
Yes	2 (10)	2 (6)	
MVPA time proportion (%)	0.16 ± 0.04	0.15 ± 0.04	0.221
MVPA time proportion (%)			0.408
< 16%	10 (48)	10 (32)	
≥ 16%	11 (52)	21 (68)	

[a] Median time of children with normal weight spent on television was used as the cutoff. Abbreviation: SD: standard error; MVPA: moderate-to-vigorous physical activity. Bold indicates statistically significant at the *p*-value < 0.05 level.

Supplement Table S3. Comparison of diet and physical activity by phone time levels.

Variables	Less phone time [a] (N = 29)	More phone time (N = 23)	p-value
	Mean ± SD or N (%)	Mean ± SD or N (%)	
Dietary intake			
Energy (kcal)	1847.29 ± 564.99	2088.75 ± 458.96	0.095
Protein (g)	77.76 ± 37.65	91.10 ± 23.88	0.127
Protein intake proportion (%)			0.208
< 17.6%	20 (69)	11 (48)	
≥ 17.6%	9 (31)	12 (52)	
Carbohydrates (g)	262.84 ± 69.34	281.51 ± 81.70	0.387
Fat (g)	57.08 ± 24.89	69.66 ± 22.30	0.061
Dietary behavior			
Average duration per meal (min)	12.24 ± 4.24	14.74 ± 4.80	0.056
Average edible weight of food consumed per meal (g)	333.26 ± 95.71	381.35 ± 96.68	0.080
Average eating speed per meal (g/min)	29.54 ± 11.31	27.89 ± 10.34	0.586
Proportion of meals with screen (%)	0.16 ± 0.17	0.31 ± 0.25	**0.015**
Proportion of meals with social interaction (%)	0.84 ± 0.19	0.84 ± 0.14	0.922
Sedentary behavior			
Sedentary behavior (min/day)	446.49 ± 75.50	476.53 ± 48.53	0.089
Physical activity			
Physical activity time (min/day)	249.76 ± 57.59	257.95 ± 47.30	0.576
MVPA time (min/day)	40.72 ± 15.90	39.07 ± 11.61	0.668
MVPA time ≥ 1h/day (%)			0.621
No	26 (90)	22 (96)	
Yes	3 (10)	1 (4)	
MVPA time proportion (%)	0.16 ± 0.04	0.15 ± 0.03	0.382
MVPA time proportion (%)			0.440
< 16%	13 (45)	7 (30)	
≥ 16%	16 (55)	16 (70)	

[a] Median time of children with normal weight spent on phone was used as the cutoff. Abbreviation: SD: standard error; MVPA: moderate-to-vigorous physical activity. Bold indicates statistically significant at the p-value < 0.05 level.

Supplement Table S4. Comparison of diet and physical activity by computer time levels.

Variables	Less computer time [a] (N = 25)	More computer time (N = 27)	*p*-value
	Mean ± SD or N (%)	Mean ± SD or N (%)	
Dietary intake			
Energy (kcal)	1823.69 ± 530.2	2074.83 ± 509.7	0.088
Protein (g)	79.96 ± 33.9	87.08 ± 31.8	0.439
Protein intake proportion (%)			0.819
< 17.6%	14 (56)	17 (63)	
≥ 17.6%	11 (44)	10 (37)	
Carbohydrates (g)	251.64 ± 69.48	289.12 ± 76.42	0.070
Fat (g)	58.30 ± 23.82	66.66 ± 24.65	0.219
Dietary behavior			
Average duration per meal (min)	13.20 ± 5.63	13.47 ± 3.55	0.837
Average edible weight of food consumed per meal (g)	342.59 ± 104.83	365.58 ± 92.24	0.407
Average eating speed per meal (g/min)	29.71 ± 14.41	27.97 ± 6.04	0.579
Proportion of meals with screen (%)	0.20 ± 0.19	0.24 ± 0.25	0.517
Proportion of meals with social interaction (%)	0.78 ± 0.19	0.89 ± 0.12	**0.023**
Sedentary behavior			
Sedentary behavior (min/day)	440.51 ± 70.20	477.61 ± 57.79	**0.044**
Physical activity			
Physical activity time (min/day)	239.58 ± 51.37	266.16 ± 52.05	0.070
MVPA time (min/day)	40.95 ± 16.09	39.10 ± 12.12	0.643
MVPA time ≥ 1h/day (%)			0.548
No	22 (88)	26 (96)	
Yes	3 (12)	1 (4)	
MVPA time proportion (%)	0.17 ± 0.04	0.15 ± 0.03	0.051
MVPA time proportion (%)			**0.027**
< 16%	14 (56)	6 (22)	
≥ 16%	11 (44)	21 (78)	

[a] Median time of children with normal weight spent on computer was used as the cutoff. Abbreviation: SD: standard error; MVPA: moderate-to-vigorous physical activity. Bold indicates statistically significant at the *p*-value < 0.05 level.

Supplement Table S5. Comparison of diet and physical activity by tablet time levels.

Variables	Less tablet time [a] (N = 28)	More tablet time (N = 24)	*p*-value
	Mean ± SD or N (%)	Mean ± SD or N (%)	
Dietary intake			
Energy (kcal)	1831.47 ± 460.73	2097.15 ± 577.72	0.077
Protein (g)	78.52 ± 28.39	89.66 ± 36.80	0.234
Protein intake proportion (%)			1.000
< 17.6%	17 (61)	14 (58)	
≥ 17.6%	11 (39)	10 (42)	
Carbohydrates (g)	250.46 ± 63.10	295.17 ± 81.45	**0.034**
Fat (g)	60.22 ± 23.50	65.47 ± 25.58	0.448
Dietary behavior			
Average duration per meal (min)	11.87 ± 3.91	15.06 ± 4.87	**0.014**
Average edible weight of food consumed per meal (g)	344.11 ± 97.09	366.69 ± 100.16	0.415
Average eating speed per meal (g/min)	31.58 ± 12.75	25.57 ± 6.96	**0.037**
Proportion of meals with screen (%)	0.23 ± 0.23	0.22 ± 0.22	0.899
Proportion of meals with social interaction (%)	0.81 ± 0.16	0.88 ± 0.16	0.131
Sedentary behavior			
Sedentary behavior (min/day)	440.09 ± 61.20	482.74 ± 65.39	**0.020**
Physical activity			
Physical activity time (min/day)	253.51 ± 54.80	253.23 ± 51.85	0.985
MVPA time (min/day)	41.07 ± 15.30	38.74 ± 12.67	0.550
MVPA time ≥ 1h/day (%)			0.615
No	25 (89)	23 (96)	
Yes	3 (11)	1 (4)	
MVPA time proportion (%)	0.16 ± 0.04	0.15 ± 0.04	0.486
MVPA time proportion (%)			0.322
< 16%	13 (46)	7 (29)	
≥ 16%	15 (54)	17 (71)	

[a] Median time of children with normal weight spent on tablet was used as the cutoff. Abbreviation: SD: standard error; MVPA: moderate-to-vigorous physical activity. Bold indicates statistically significant at the *p*-value < 0.05 level.
